# Novel impact of the DNMT3A R882H mutation on GSH metabolism in a K562 cell model established by TALENs

**DOI:** 10.18632/oncotarget.16449

**Published:** 2017-03-22

**Authors:** Li Yang, Ya’Nan Liu, Na Zhang, Xiao’Yi Ding, Wei Zhang, Ke’Feng Shen, Liang Huang, Jian’Feng Zhou, Sen Cui, Zun’Min Zhu, Zheng Hu, Min Xiao

**Affiliations:** ^1^ Department of Hematology, Tongji Hospital Affiliated with Tongji Medical College, Huazhong University of Science and Technology, Wuhan, Hubei Province, P.R.China; ^2^ Department of Hematology, Zhongnan Hospital of Wuhan University, Wuhan, Hubei Province, P.R. China; ^3^ Department of Hematology, Xijing Hospital Affiliated by The Fourth Military Medical University (FMMU), Xi'an, Shanxi Province, P.R.China; ^4^ Department of Obstetrics and Gynecology, Tongji Hospital, Tongji Medical College, Huazhong University of Science and Technology, Wuhan, Hubei, China; ^5^ Department of Obstetrics and Gynecology, The First Affiliated Hospital, Sun Yat-Sen 14 University, Guangzhou, Guangdong, China; ^6^ Qinghai University, XiNing, Qinghai Province, P.R.China; ^7^ Department of Hematology, Qinghai University Affiliated Hospital, Qinghai University, XiNing, Qinghai Province, P.R.China; ^8^ Department of Hematology, Henan Provincial People’s Hospital, ZhengZhou, Henan Province, P.R.China

**Keywords:** DNMT3A R882H mutation, TALENs, K562, GSH, SLC7A11

## Abstract

DNA methyltransferase 3A (DNMT3A) mutations occurred in 18%~23% of acute myeloid leukemia (AML) patients, and were considered to be an adverse prognostic factor for adult de novo AML cases. However, the relevant molecular mechanism of the mutation in AML pathogenesis remains obscure. In this study, we established K562 and SKM1 cell model carrying the DNMT3A R882H mutation via transcription activator-like effector nuclease (TALEN) and Clustered regularly interspaced short palindromic repeats (CRISPR/Cas9) technology, and discovered that mutated DNMT3A could promote the proliferative capability of malignant cell clones. Further RNA microarray analysis revealed that some genes crucial for glutathione (GSH) synthesis, including CTH, PSPH, PSAT1 and especially SLC7A11 (the cysteine/glutamate transporter) were significantly up-regulated, which resulted in significant elevation of intracellular GSH levels. A subsequent experiment demonstrated that the mutant clones are resistant to chemotherapy as well as SLC7A11-inhibitorsBy shRNA induced SLC7A11 silencing, we discovered profoundly decreased cellular GSH and cell proliferative ability of DNMT3A mutated clones. Our results provided novel insight into the role of the DNMT3A R882H mutation in AML pathogenesis and suggested that targeting the cellular GSH synthetic pathway could enhance the current therapy for AML patients with the DNMT3A R882H mutation.

## INTRODUCTION

DNA methylation plays a major role in epigenetic modifications. It not only has a fundamental impact on physiological processes such as gene regulation, chromatin structure stability, embryonic development, X chromosome inactivation and genomic imprinting in normal cells [1–4] but also has substantial influences on cell transformation and carcinogenesis [5, 6]. DNA methylation results from the activity of a family of enzymes called DNA methyltransferases (DNMTs), of which DNA methyltransferase 3A (DNMT3A) functions primarily to initiate *de novo* DNA methylation by catalyzing the addition of a methyl group to the cytosine residue of CpG dinucleotides and thus plays an important role in the epigenetic regulation of genes [1, 7]. Acute myeloid leukemia (AML) is a genetically heterogeneous hematologic malignancy characterized by the clonal expansion of myeloid blasts. In recent years, *DNMT3A* mutation has recently been shown to predict an inferior prognosis in AML [8–13].

DNMT3A mutation is detected in approximately 18%~23% newly diagnosed AML patients [13–17], while the mutation is less frequently detected in other hematological malignancies [18–21]. Although DNMT3A mutations affecting many different sites in the coding region have been described, mutation of the amino acid Arg882 (R882) within the catalytic domain of DNMT3A is the most common type of DNMT3A mutation. [13, 16, 17]. For the past several years, efforts have been made to explore the functional consequence associated with DNMT3A mutation. The DNMT3A R882H mutation gave rise to decreased DNMT3A enzymatic activity *in vitro*, which suggests that the mutation causes loss of function of DNMT3A [16, 22]. Furthermore, another *in vitro* experiment showed that murine DNMT3A with the R878H mutation, which corresponds to human DNMT3A R882H, failed to mediate DNA methylation; the mutation further interfered the methylation capability of wild-type DNMT3A in murine embryonic stem (ES) cells, suggesting the dominant negative role of the DNMT3A R882H mutation [23]. As for *in vivo* studies, by using a retroviral transduction and bone marrow transplantation (BMT) approach, a recent study discovered that the DNMT3A R882H mutation induced aberrant hematopoietic stem/progenitor cell proliferation and culminated in chronic myelomonocytic leukemia-like disease at 12 months post BMT in all the transplanted mice [24]. In addition, another animal experiment showed that mice with the conditional ablation of DNMT3A in hematopoietic stem cells (HSCs) led to an impairment of HSC differentiation and abnormal expansion of HSCs in the bone marrow [25]. Collectively, these data have underscored a critical role of the DNMT3A R882H mutation in leukemogenesis.

Although current studies have made great efforts to clarify the epigenetic landscape alterations modified by mutated DNMT3A [24, 25], the advances are far from satisfactory in unraveling the association between DNMT3A mutation and the inferior clinical outcomes in AML patients, which limited the development of novel targeted therapy against the mutation. Therefore, novel strategies are urgently needed to establish a cellular model of DNMT3A mutation with a comparable genetic background.

Currently, genome-editing technologies such as transcription activator-like effector nucleases (TALENs) and Clustered regularly interspaced short palindromic repeats (CRISPR/Cas9) have been widely used in cancer research [26, 27]. In this study, we established a K562 / SKM1 cell line with the DNMT3A R882H mutation using TALENs / CRISPR-Cas9 technology to evaluate the impact of the DNMT3A R882H mutation on cellular functions of malignant clones and to gain insight into the potential molecular mechanism.

## RESULTS

### Generation of isogenic K562 clones with the DNMT3A R882H mutation using designed TALENs

**Figure 1:**
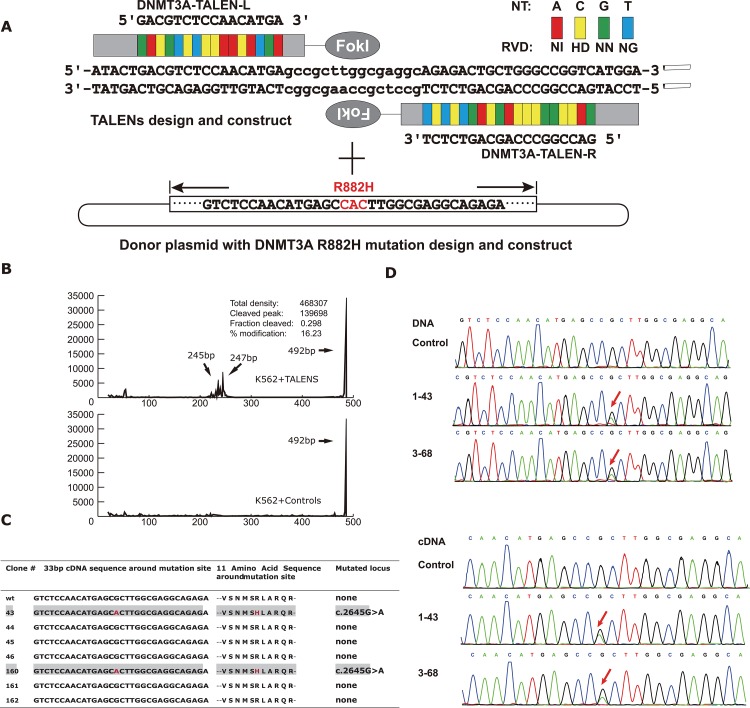
**(A)** Schematic of system for establishing K562 cell line with DNMT3A R882H mutation by TALENs. After selection of TALENs target site and assembly of TALEN plasmids, one donor plasmid with DNMT3A R882H mutation was designed and constructed. Both of the TALENs plasmid and donor plasmid were electroporated into K562 cells. Then using fluorescence-activated cell sorting to sort the cells into single cell in 96-well plates. After 2 weeks, pick up the colonies that had expanded to enough numbers. Isolate DNA from the colonies as template for PCR amplification and confirm the mutation with Sanger sequencing to distinguish the mutant clones with wile-type ones. **(B)** Using capillary electrophoresis to estimate gene modification levels of the TALENs by T7 Endonuclease I assay. **(C)** Summary of Sanger sequencing results of 33bp around DNMT3A mutation locus and corresponding amino acid changes from positive isogenic mutant clones derived from K562 cell line transfected with TALENs. **(D)** The sequencing results of the mutant clones compared with wild-type ones. Using genomic DNA and cDNA as template for PCR amplification respectively. After ligating PCR product with T-vector, confirm the sequence with Sanger sequencing. The red arrow shows the position of DNMT3A R882H mutation (CGC→CAC).

To generate isogenic K562 clones with the DNMT3A R882H mutation, we designed a pair of TALENs specifically targeting the DNMT3A R882 site. The TALEN target site was chosen within 50 bp around the R882 site in the DNMT3A exon 23 coding sequence. the TALENs comprised 16 and 19 repeats of the cleavage site with a 15 bp spacer (Figure 1A).

To evaluate the nuclease activity of our designed TALENs at the intended target, a T7E1 mismatch sensitive assay was performed, and the T7E1 digested products were scanned through capillary electrophoresis. As shown in Figure 1B, we confirmed that the activity of the DNMT3A-TALEN plasmid pairs was efficient in K562 cells, as the modification ratio reached 16.23% compared to the control, allowing for subsequent screening of K562 clones with genetically edited DNMT3A.

Next, to achieve successful TALEN genetic editing, which depends on a homologous derived recombination (HDR) event at the double-strand break (DSB) site, one donor plasmid with the DNMT3A R882H mutation (CGC>CAC, red characters in Figure 1A) with homology to the region was designed and constructed. Subsequently, we co-transfected both the DNMT3A-TALEN plasmids and the donor plasmid into K562 cells and seeded the cells at a low density in a 96-well plate for recovery and homogeneous expansion.

After the isolation of each clonal cell population, we performed genomic DNA extraction and PCR amplification, followed by Sanger sequencing to identify the specific HDR characteristics. Ultimately, we obtained two clones with the DNMT3A R882H mutation from a total of 160 clones (2/160, 1.25%) and verified that their DNA and mRNA sequences were consistent with the mutant region of the donor plasmid (Figure 1C, 1D). In addition, one wild-type clone derived from the same screening procedure was randomly selected as a negative control.

As for the assessment of off-target gene effects of the designed TALENs, Paired Target Finder maintained by TAL Effector Nucleotide Targeter 2.0 (https://tale-nt.cac.cornell.edu/node/add/talef-off-paired) was applied [28, 32]. We identified 28 potential off-target sites in the human genome, which are listed in Supplementary Table 1S. To determine whether the TALENs would bind to these sites and introduce unwanted genomic aberrations, we performed whole-exome sequencing on parent K562 cells, wild-type clones and mutant clones. As a result, no modifications were observed in the sequences of these 28 sites in mutant clones.

Taken together, our results suggested that this pair of TALEN plasmids we constructed were specific to the DNMT3A gene and that the K562 cell line with the DNMT3A R882H mutation was successfully established.

### Generation of SKM1 cell model carrying DNMT3A R882H heterozygous mutation by CRISPR-Cas9 system

Since K562 cell line harbors BCR-ABL fusion gene, and the existence of BCR-ABL transcript could potentially deviate the impacts of DNMT3A mutation on gene expression. Therefore, another cell model carrying DNMT3A R882H was established based on SKM-1 cell line by CRISPR-Cas9 system.

As a result, after analyzing on a total of 450 clones, we successfully acquire 2 clones which harbors heterozygous mutation of DNMT3A R882H, yielding 0.4% heterozygous mutation generation. To further screen undesired off-target effects, the top five predicted off-targets sites was detected by Sanger sequencing and no sequence alterations were detected, indicating high specificity of CRISPR/Cas9 system applied in this study.

### The DNMT3A R882H mutation promotes K562 cell proliferation

**Figure 2:**
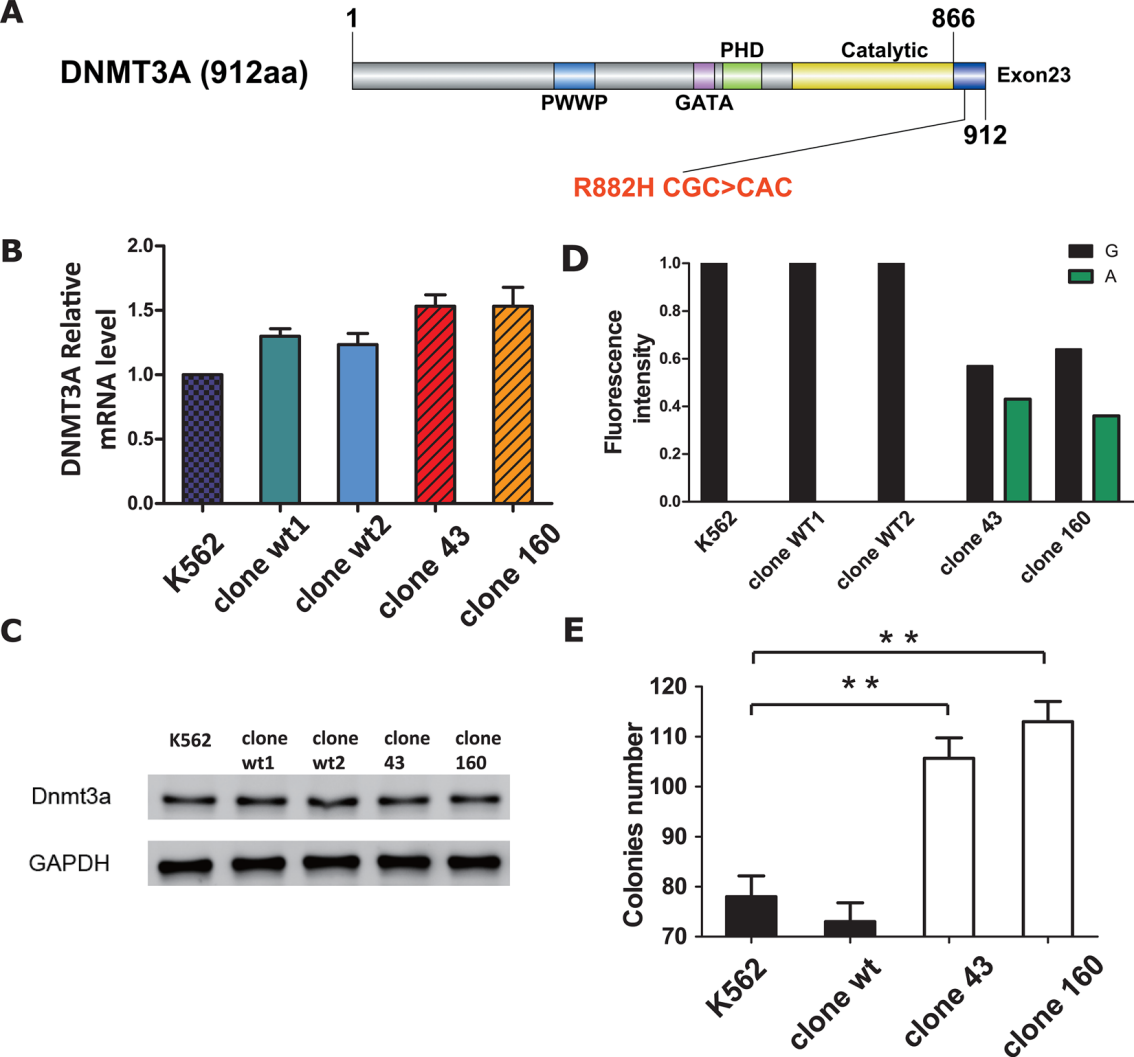
**(A)** Schematic drawing of the architecture of DNMT3A protein and the letters in red indicate the position of R882H mutation (CGC>CAC). **(B)** RT-PCR results of DNMT3A mRNA expression in parent K562 cell line, wild-type clone and mutant clones. Statistical significance was analyzed using one-way ANOVA followed by Bonferroni test (*P<0.05, **P<0.01). **(C)** Protein expression of DNMT3A in parent K562 cell line, wild-type clone and mutant clones respectively. β-actin was used as positive control. **(D)** Quantification of DNMT3A wild-type allele **(G)** and R882H mutant allele **(A)** expression by fluorescence intensity after RT-PCR. The respective height of each column (black column indicated G and green column indicated A respectively) was determined from their corresponding fluorescence intensity. **(E)** Number of colonies formed. Cells were counted after plating in Methyl cellulose medium one week. Error bar represent SEM. Statistical significance was analysed using one-way ANOVA followed by Bonferroni test (*P<0.05, **P<0.01).

As DNMT3A R882 is located in the catalytic domain of the protein (Figure 2A), the putative dominant negative role of the DNMT3A R882H mutant protein invoked many researchers worldwide to investigate its role in AML pathogenesis and disease progression. In this study, we aimed to unravel the biological as well as the molecular impact of the mutation based on our established K562 DNMT3A R882H mutant cell line.

First, we aimed to investigate the impact of the R882H mutation on DNMT3A gene transcription. To exclude the possibility of clonal variation, 2 different clones of wild-type control cell clones were selected. The overall expression of DNMT3A mRNA and protein in five cell lines (normal K562 cells, wild-type clone 1, wild-type clone 2, mutation clone 43 and mutation clone 160) was detected via RT-PCR and Western Blot techniques. As a result, we found that both the mRNA and protein levels of DNMT3A were slightly and evenly elevated in DNMT3A mutant and wild-type clones compared to the levels in normal K562 cells (Figure 2B, 2C), indicating that the DNMT3A R882H mutation had a limited influence on DNMT3A gene expression. In addition, we quantified the DNMT3A R882H mutant allele burden by using cDNA from five cell lines. The results showed that the levels were comparable between wild-type and mutant DNMT3A transcripts for both mutant clones 43 and 160 (G 57% VS A 43% in mutant clone 43; G 64% VS A 36% in mutant clone 160, Figure 2D), ruling out the possibility of a haploinsufficiency effect of the DNMT3A R882H mutation.

Next, to evaluate the effect of the DNMT3A R882H mutation on K562 cell growth, a colony forming assay was conducted for all four cell lines. Surprisingly, after incubation for one week, the number of colonies of the mutant clones had increased significantly compared to that of the wild-type clone and of normal K562 cells (105 and 113 colonies on average for mutant clones 43 and 160, respectively, VS 78 and 73 colonies on average for the wt clone and normal K562 cells, respectively; p=0.0038 for clone 160 VS normal K562, p=0.0091 clone 43 VS normal K562, Figure 2E). Meanwhile, the colony numbers of the wild-type clone and normal K562 cells exhibited non-significant differences (p=0.4245). In addition, the effects of the mutation on apoptosis and the cell cycle were also tested, but there was no significant difference between the mutant clones and normal K562 cells (data not shown).

### TALEN-mediated DNMT3A R882H mutation leads to multiple changes in the gene expression signature in K562 cells

**Figure 3:**
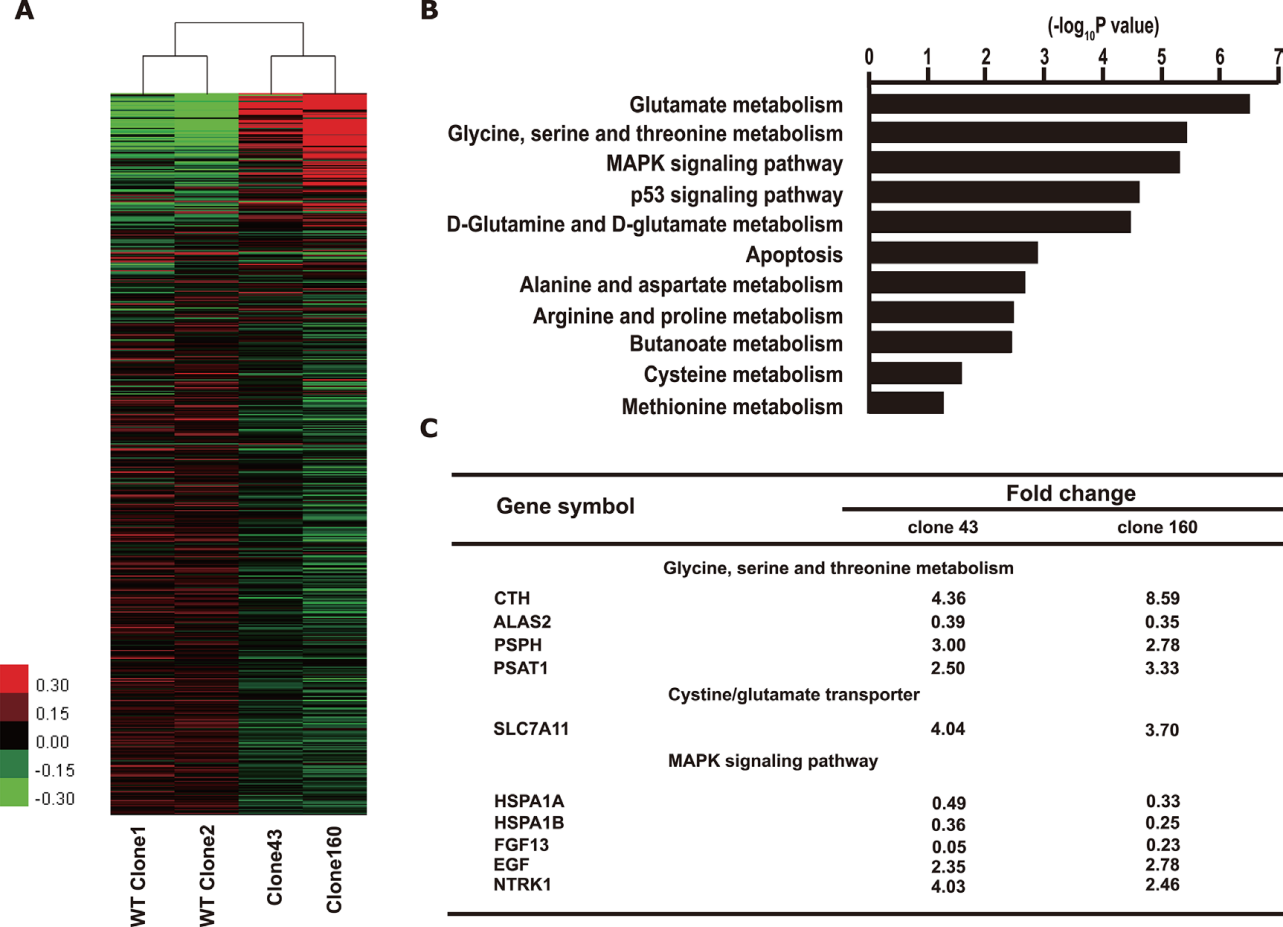
Expression profiling of K562 clones with DNMT3A R882H mutation **(A)** Heat map of microarray data shows different gene expression pattern between clones of mutant and wild-type. Red indicates relatively up-regulated genes. Green indicates relatively down-regulated genes. **(B)** Functional clustering analysis from the microarray data using KEGG database. **(C)** Relative expression changes of genes in amino acid metabolic/transport and MAPK pathways from K562 clones with DNMT3A R882H mutation (Clone 43 and Clone 160) compared with WT clones.

In order to explain the proliferation differences between mutant and wild-type cell lines, we compared the global gene expression profiles among 2 DNMT3A R882H mutation cell lines (mutant clones 43 and 160) and 2 wild-type clones by oligonucleotide microarray analysis. Unsupervised cluster analysis on the whole transcriptomes of the 4 clones indicated significant distinctions in the expression patterns between the mutant clones and their wild-type counterparts. Additionally, nonsignificant differences in the expression pattern were observed within the mutant group (clone 43 VS clone 160) and the wild-type group (WT clone 1 VS WT clone 2) (Figure 3A). As the same genome-editing procedure was conducted on all 4 clones, it is reasonable to believe that such gene expression changes between the mutant and wild-type clones resulted from the TALEN-mediated gene mutation rather than from clonal deviation. Thus, it is reasonable to explore gene expression alterations that are specifically associated with the DNMT3A R882H mutant by using the current cell model.

Next, gene functional enrichment analysis was performed on all the differentially expressed genes (DEGs) that exhibited ≥2-fold change compared with wild-type clones (Figure 3B). KEGG pathway clustering showed that the most prominent pathways that were altered by DNMT3A R882H mutation included the amino acid metabolism, MAPK, apoptosis and p53 signaling pathways.

In detail, some classical genes in the MAPK pathway were found to be up-regulated, such as EGF (fold changes of 2.35 and 2.78 in the 2 mutant clones, respectively) and NTRK1 (fold changes of 4.03 and 2.46 in the 2 mutant clones, respectively), which is a tyrosine kinase receptor family member. However, other components exhibited down-regulation, such as FGF13, HSPA1A, and HSPA1B, rendering the exact role of the MAPK signaling cascade in mutated clones unclear.

**Figure 4:**
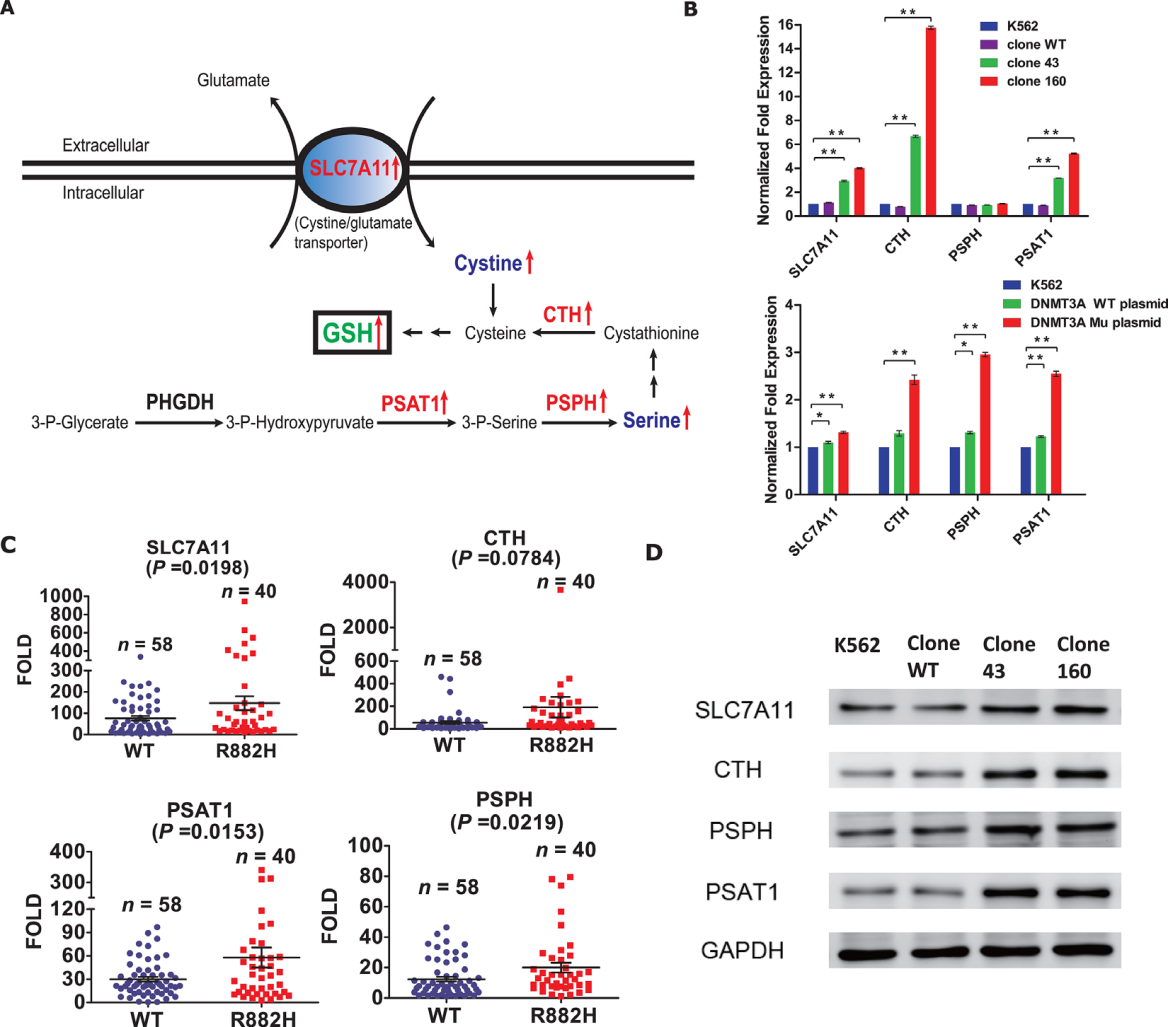
**(A)** Schematic of metabolic pathways potentially affected by DNMT3A R882H mutation in K562 clones. Enzymes (corresponding mRNA) that were up-regulated are displayed in red. Metabolites that were up-regulated are showed in blue. Green font indicates consequently increased GSH level. (SLC7A11, the cysteine/glutamate transporter; CTH, cystathionase; PSPH, phosphoserine phosphatase; PSAT1, phosphoserine aminotransferase 1; GSH, glutathione). **(B)** RT-PCR validations of target genes differentially expressed in the microarray results. The upper diagram shows SLC7A11, CTH, PSPH and PSAT1 mRNA levels in normal K562 cell line, wild-type clone and mutant clones respectively. The lower diagram shows SLC7A11, CTH, PSPH and PSAT1 mRNA levels in normal K562 cell line, K562 transfected with DNMT3A wild-type (WT, without DNMT3A R882H mutation) plasmid and K562 transfected with DNMT3A mutant (Mu, with DNMT3A R882H mutation) plasmid. Statistical significance was analysed using two-way ANOVA followed by Bonferroni test (*P<0.05, **P<0.01). **(C)** SLC7A11, CTH, PSPH and PSAT1 mRNA levels in 40 DNMT3A R882H mutated AML patients (R882H) and 58 DNMT3A wild-type AML patients (WT) were validated by using RT-PCR. Each symbol on the scatter plot represents the average value analysed on individual sample from three independent experiments. Statistical differences between two groups were analysed using unpaired t test. **(D)** Protein expressions of SLC7A11/CTH/PSPH/PSAT in parent K562 cell line, wild-type clone and mutant clones respectively were detected by western blot. β-actin was used as positive control.

What intrigued us the most was the discovery that a series of genes that participate in the amino acid metabolic pathways were significantly up-regulated (Figure 3C). Moreover, the KEGG database indicated that the enzymes coded by these genes collaboratively contributed to the synthetic process of cellular GSH, as shown in Figure 4A. Specifically, the expression of two key enzymes, phosphoserine phosphatase (PSPH) and phosphoserine aminotransferase 1 (PSAT1), increased significantly, which would lead to elevated levels of serine as well as of its downstream product, cystathionine. Cystathionine is an intermediate product in the synthesis of cysteine that can be cleaved into cystine and α-ketobutyrate by cystathionine gamma-lyase (CTH). Meanwhile, the elevated expression of the cystine/glutamate transporter (SLC7A11) can promote the cellular uptake of cystine, which would also result in higher levels of cysteine because intracellular cystine could be reduced to cysteine rapidly. Ultimately, with the profoundly expanded amount of cysteine as an indispensable ingredient, the intracellular GSH levels would be significantly increased.

### Validation of four target genes altered by the DNMT3A R882H mutation identified by microarray analysis

To validate the microarray results, we first used RT-PCR and Western Blot methods to detect the mRNA and protein levels of these four target genes in the four clones directly. Except for PSPH, the mRNA levels of other three target genes were consistent with the microarray data. For clone 160, we observed the significant up-regulation of SLC7A11 (fold change 4.02, p<0.01), CTH (fold change 15.77, p<0.01) and PSAT1 (fold change 5.23, p<0.01); for clone 43, the up-regulation was less obvious but was also significant (Figure 4B upper). For protein expression, Western Blot analysis indicated that all four target genes exhibited various degrees of up-regulation in the mutant clones compared with the normal K562 cells, while the wild-type clones showed non-significant changes (Figure 4D).

Next, to confirm the effect of the DNMT3A R882H mutation on the four target genes, we electroporated the human DNMT3A gene expression plasmid (wild-type or DNMT3A R882H mutation) into normal K562 cells. The results of RT-PCR showed that although the clones transfected with the mutant and wild-type plasmids both exhibited elevated mRNA levels of all four target genes, the elevation in expression was much more significant in the clones transfected with the mutant plasmid. The results above verified our hypothesis that the DNMT3A R882H mutation affected the expression of these four target genes involved in the process of GSH synthesis (Figure 4B lower).

To exclude the effects of BCR-ABL gene transcript which originally existed in K562 cell model on GSH related gene expression, we further detected four target gene expression on our SKM1 DNMT3A R882H cell model. As a result, comparing with SKM1 and wild-type control clones, both DNMT3A R882H mutated SKM1 clones exhibited significantly elevated expression of all four target genes (Supplementary Figure 1C). Our result confirmed the role of DNMT3A R882H mutation on promoting expression of genes in GSH synthetic pathway

In addition, we further validated the results by conducting RT-PCR on clinical samples from AML patients treated in our hospital. mRNA samples from 40 AML patients with the DNMT3A R882H mutation, together with 58 AML patients with wild-type DNMT3A were randomly selected and subjected to subsequent analyses. The results showed that the expression of three of the target genes (SLC7A11, PSPH, PSAT1) were significantly increased in patients with the DNMT3A R882H mutation compared with those with wild-type DNMT3A (Figure 4C). However, the expression of CTH was not significantly different between the two groups (p = 0.0784). In general, our results suggested that the DNMT3A R882H mutation indeed enhanced the expression of enzymes in the GSH synthesis pathway at both the mRNA and protein levels. However, it remains unclear whether cellular GSH production was actually altered in DNMT3A R882H mutant clones, and more experiments to unravel the downstream biological functions remain needed.

### DNMT3A R882H mutation induces aberrations of the intracellular GSH level and enhances leukemic clone survival and drug tolerance

**Figure 5:**
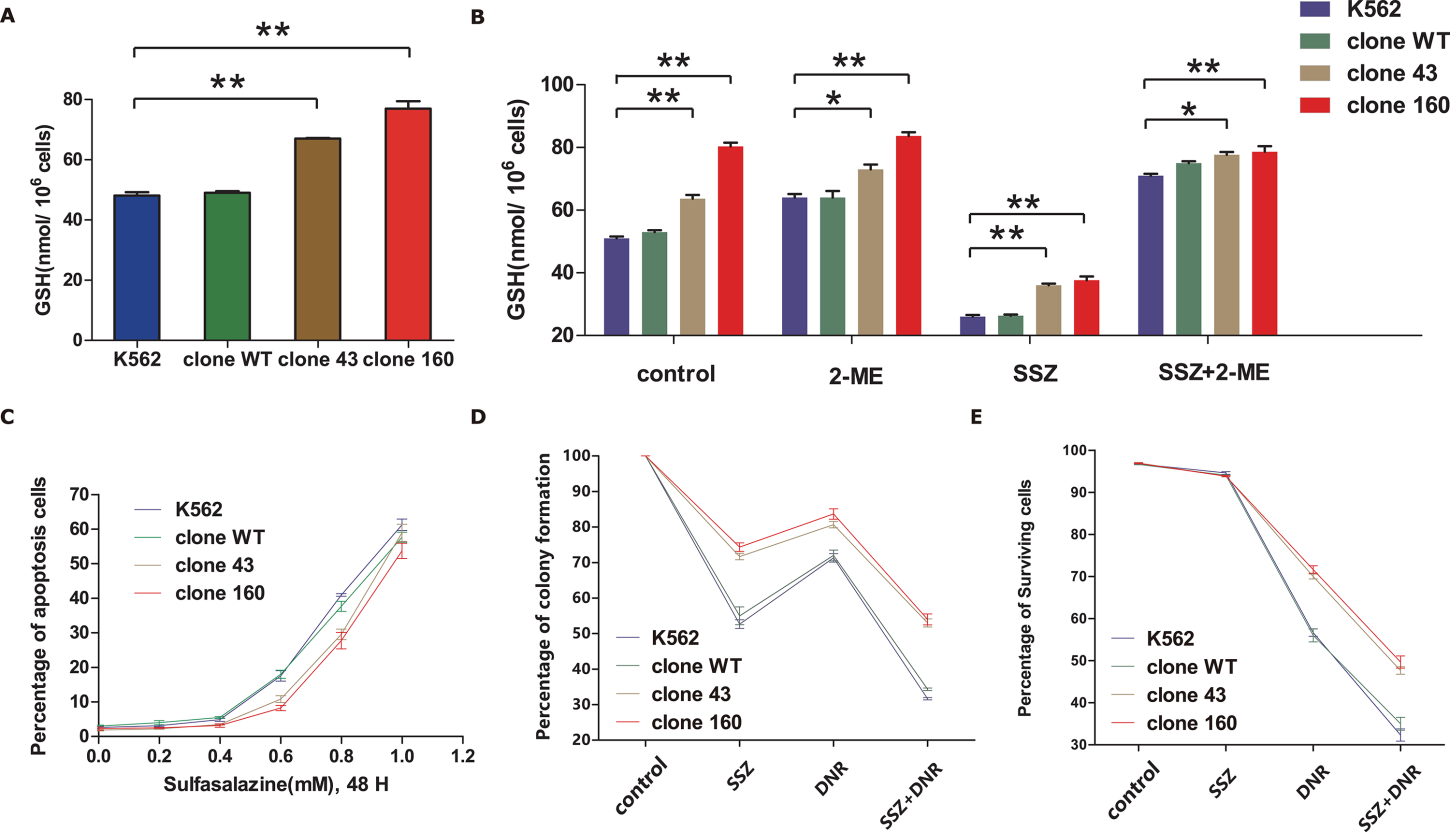
**(A)** Diagram of Glutathione (GSH) level of normal K562 cell line, wild-type clone and mutation clones. The average GSH level from three independent experiments were statistically compared by two-way ANOVA followed by Bonferroni test (*P<0.05, **P<0.01). **(B)** Glutathione (GSH) content of normal K562 cell line, wild-type clone and mutantion clones at normal state and treated with SSZ (0.3mM) /2-ME (66uM) alone or combined utilization of both drugs for 48H. The average GSH level from three independent experiments were statistically compared by two-way ANOVA followed by Bonferroni test (*P<0.05, **P<0.01). **(C)** Assessment of pro-apoptotic effect of SSZ on all four clones by incubating the cells with SSZ over a range of 0-1.0 mM for 48 hours incubation time. Cell viability was determined by using flow cytometry to detect labelled Annexin V-FITC/PI. **(D)** Effect of SSZ and DNR on colony formation in parent K562 cell line, clone WT, clone 43 and 160. Cells were pre-incubated with 0.3 mM SSZ for 24 h. The drug was then removed and the cells was further cultured with 50 nM DNR for 24 h. Colonies were enumerated after about 2 weeks. Error bar represent SEM. Statistical significance was analyzed using a t test (*P < 0.05). **(E)** Comparisons of the effects of SSZ, DNR alone and combinations of two of them and all three ones on viability of parent K562 cell line, wild-type clone and mutant clones. Cells were treated with SSZ (0.3mM) and DNR (200nM) alone or combined for 48H. Cell viability was determined by using flow cytometry to detect labelled with Annexin V-FITC/PI. Results are the average of three independent experiments. Asterisks indicate significant difference (*P<0.05).

Based on the above questions, we detected intracellular GSH levels in all four clones by using a GSH assay, as described in methods section. The result revealed that compared with normal K562 cells, the mutant clones (clone 43 and clone 160) contained significantly higher levels of intracellular GSH (with average fold changes of 1.39 and 1.60, respectively), while the wild-type clone exhibited insignificant alterations (Figure 5A). The results confirmed our hypothesis that the up-regulation of the four target genes in the GSH metabolic pathway could result in an elevated intracellular GSH content.

As is well known, GSH is a reducing agent that plays an important role in cell resistance to radiation and drug-induced cytotoxicity. Therefore, cellular GSH synthetic machinery is a fascinating target in cancer therapy. Because SLC7A11 played a fundamental role in the accumulation of intracellular cystine for GSH production, we planned to evaluate the efficacy on the inhibition of GSH production as well as the therapeutic applicability of blocking the SLC7A11 transporter in mutant AML clones. Previous studies have reported that sulfasalazine (SSZ) is a potent and specific inhibitor of SLC7A11 that can inhibit cystine uptake and lead to GSH depletion in cells [33]. 2-Mercaptoethanol (2-ME), however, allows for the cellular uptake of cysteine via the leucine transporter in the form of a 1-ME-cysteine mixed disulfide at ~60 μM [34], which counteracts the effects of SSZ. Therefore, we performed a GSH assay on all four clones pre-treated with SSZ/2-ME alone or in combination.

Because the cell viability is vital for GSH measurement, the most appropriate concentration of SSZ (0.3 mM, similar to the content in patients’ sera) was selected in order to influence the intracellular GSH content most significantly while minimally affecting cell viability. Next, using untreated cells as the control group, all four clones were treated with 0.3 mM SSZ / 60 μM 2-ME alone or in combination. As shown in Figure 5B, under the treatment of 0.3 mM SSZ, GSH levels dropped by approximately 50% in all four clones. However, the mutant clones (clones 43 and 160) still exhibited significantly higher levels of GSH compared with normal K562 cells. Meanwhile, when 2-ME was added, with or without SSZ treatment, the GSH levels remained steady and even slightly increased compared with those of the control groups. These results indicated that the decrease of intracellular GSH levels was due to the specific inhibition of SLC7A11, which could be reversed by 2-ME treatment.

**Figure 6:**
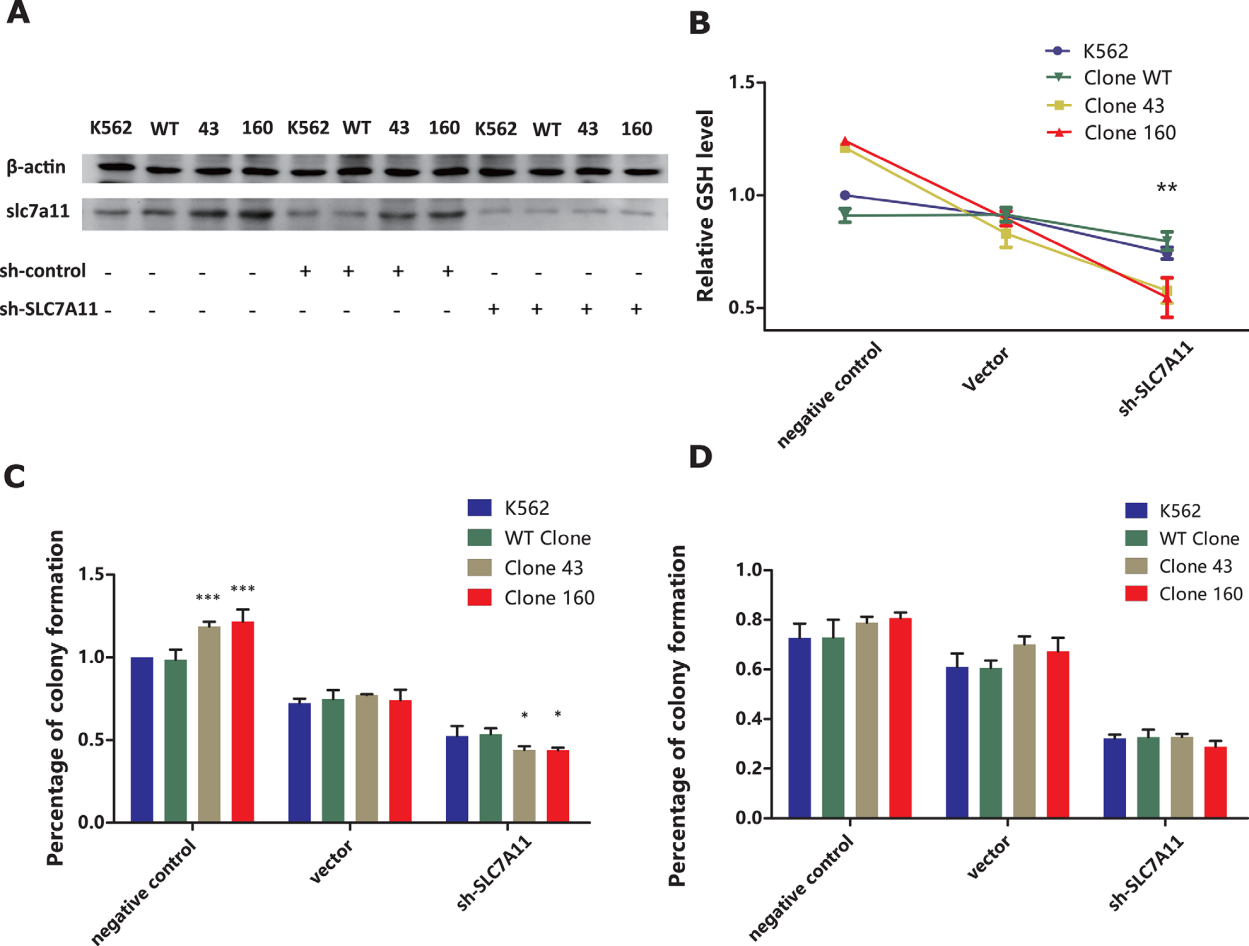
**(A)** Western Blot analysis on slc7a11 protein expression of K562/WT clone/Clone 43/Clone 160 cell line transfected with or without lentivirus mediated SLC7A11 RNAi vector and negative control vectors **(B)** Comparison of the negative/sh-control/RNAi treatment group on cellular GSH level in K562/WT clone/Clone43/Clone160 cell lines. GSH level was measured 48h after transfection. Average GSH level from three independent experiments were normalized and statistically compared by two-way ANOVA followed by Bonferroni test (*P<0.05, **P<0.01). **(C)** Effect of negative/sh-control/RNAi treatment on colony formation in parent K562 cell line, clone WT, clone 43 and 160. Cells were incubated in 96-well plate after 48h of transfection treatment and colonies were enumerated after about 2 weeks. Error bar represent SEM. Statistical significance was analyzed using two-way ANOVA followed by Bonferroni test (*P< 0.05, ***P<0.001). **(D)** Combination of DNR and negative/sh-control/RNAi treatment on colony formation in parent K562 cell line, clone WT, clone 43 and 160 cell lines. After 48h incubation all four cell lines were treated with 50 nM DNR for 24 h, and then incubated in 96-well plate for 2 week for colony enumeration. Statistical significance was analyzed using two-way ANOVA followed by Bonferroni test.

To further validate the crucial role of SLC7A11 for intracellular GSH level, we managed to knock-down SLC7A11 expression by lenti-viral mediated shRNA vector transfected into all four clones. Firstly, western blot analysis confirmed that all four cell clones transfected with sh-SLC7A11 vector exhibited significantly decreased level of slc7a11 protein expression (Figure 6A). Furthermore, all four clones transfected with shRNA vector exhibited significantly drop of GSH level comparing with vector control and negative control group. Notably, clone 43 and clone 160 exhibited much more significant decreased level of GSH comparing with K562 and WT clone (Figure 6B).

Subsequently, the pro-apoptotic effect of SSZ was assessed on all four clones by incubating the cells with the drug over a range of concentrations for 48 hours incubation time (Figure 5C). The results indicated that as the concentration of SSZ increased, all four clones suffered an increased rate of apoptosis. However, although the change was not significant, at SSZ concentrations between 0.4 mM and 0.6 mM, the apoptotic rates of the mutant clones (clones 43 and 160) was lower than those of normal K562 cells and WT clones. As the above results indicated the potential therapeutic role that SSZ might possess, we further tested the efficacy on the DNMT3A R882H mutant clone by the combined use of SSZ and a regular chemotherapeutic agent, Daunorubicin. As GSH is the major cellular antioxidant and is crucial for cellular proliferation and sensitivity against cytotoxic drugs [28]. Therefore, we investigated the impact of DNR on proliferation and apoptosis among four different clones with the assistance of SSZ.

A colony forming assay was conducted on all four clones, which were pre-treated with 0.3 mM SSZ and 50 nM DNR alone or in combination. As shown in Figure 5D, when treated with SSZ or DNR alone, various degrees of proliferative inhibition were witnessed in all four clones (P<0.01, paired t test). Among them, the mutant clones (clones 43 and 160) again exhibited higher colony forming capacities compared with normal K562 cells and with the WT clone. This finding was in accordance with the higher SLC7A11 expression and intracellular GSH levels in the mutant clones. The combined regimen of the two agents in all four clones resulted in a notably higher degree of inhibition than did SSZ or DNR alone (P<0.05, paired t test). Unfortunately, the proliferative abilities of the mutant clones remained significantly higher than those of the normal K562 cells and WT clone (P<0.01, paired t test).

Then, an apoptosis assay was performed on these four clones following treatment with the same drugs, except that we adjusted the DNR concentration up to 200 nM to increase its efficacy. After 48 hours of incubation, cellular apoptosis was quantified using Annexin V and PI staining by flow cytometry. As shown in Figure 5E, the surviving percentage of mutant clones treated with DNR alone was significantly higher than that of the normal K562 cells and WT clone (P<0.05, paired t test), which was in accordance with the observation that mutant clones contained higher levels of intracellular GSH. Moreover, for combined treatment with SSZ and DNR, each of the four clones exhibited rapidly declining survival rates compared with those treated with DNR alone (P<0.01 for normal K562 cells and WT clone, P<0.001 for clones 43 and 160, paired t test), which strongly indicated that SSZ treatment sensitized mutant clones to DNR. However, despite the use of the combined regimen, AML clones bearing the DNMT3A R882H mutation remained superior in terms of their survival/proliferative capacities compared with their wild-type counterparts.

Additionally, we repeated colony forming assay on four clones treated with DNR and SLC7A11 shRNA vector transfection. As is shown in Figure 6C, mutated clones exhibited significantly higher colony forming capacity comparing with K562 and WT clone (P<0.001, 2-way ANOVA). After transfected with vector control, all four clones suffered notable decline of colony forming numbers (clone 43 77.30% / clone 160 74.1% VS K562 72.33% / WT clone 74.79%). When transfected with shRNA, mutated clone 43 and clone 160 had significantly more decline in colony forming capacity, with average colony forming level 44% and 43.8% comparing with 52.4% and 53.6% in K562 and WT clone (P<0.05, 2-way ANOVA). Furthermore, when 50 nM DNR was added in all four clones with shRNA treatment, all clones exhibited significantly drop in colony forming numbers, especially in shRNA treatment group (Figure 6D). However, when combining with DNR and shRNA treatment, mutated clone didn’t show significant difference comparing with K562 and wildtype clones (P>0.05, 2-way ANOVA).

## DISCUSSION

In this study, we utilized TALEN / CRISPR-Cas9 technology to establish a cell model carrying the DNMT3A R882H mutation. TALEN technology has many advantages over the earlier version of its kind, ZFNs. First, TALENs can be designed and constructed in as quickly as two days [28, 30]. Additionally, TALEN repeat arrays can be easily extended to an expected length. While engineered ZFNs typically bind to 9- to 18-bp sequences, TALENs are often built to bind to 18-bp sequences or longer. Another advantage of TALENs over ZFNs is that fewer constraints exist on site selection for efficient binding because multiple possible TALEN pairs are available for each bp of a random DNA sequence [30]. Indeed, compared with the recently popular CRSIPR-Cas9 system, TALENs exhibited a certain degree of complexity in their experimental procedure, but their relatively long recognition sequence was efficient in minimizing binding to off-target sites [35]. By performing whole exome sequencing, we ruled out the possible existence of all potential off-target sites. Combining the fact that the modification levels of TALENs in our experiments reached 16.23%, the results demonstrated that TALENs were an efficient and accurate tool for constructing a point mutation cell model.

In subsequent cellular functional experiments, we discovered that the mutant clones exhibited significantly promoted cellular proliferative capability. These findings were consistent with previous studies. For example, Yan XJ et al [16] found that the overexpression of DNMT3A R882H and R882C mutants in 32D cells, an interleukin-3 (IL-3)-dependent mouse myeloid cell line, could promote the proliferation of cells even without IL-3. Then, Xu J et al. [24], from the same research center, by using a murine BMT model with HSPCs transduced with DNMT3A R882H, reported that the mutation could induce the hematopoietic cells to acquire a growth advantage over residual normal blood cells at 6 mo post-BMT and 1 y post-BMT. Moreover, Shlush LI et al. [36] showed that approximately 25% of adult AML patients carried the DNMT3A mutation in pre-leukemia HSCs and that the mutation could enhance pre-leukemia HSC proliferation, which probably accounted for the clonal expansion of the pre-leukemia HSCs identified at diagnosis. Other studies using mouse model also found that HSCs lacking DNMT3A had a competitive growth advantage [25, 37].

To unravel the underlying molecular mechanism, we further performed gene expression microarray analysis of the mutated clones. Our cluster analysis showed that some genes (SLC7A11, CTH, PSPH, and PSAT1) that are crucial for GSH synthesis were up-regulated, and we subsequently confirmed that intracellular GSH levels were elevated in mutant clones. Interestingly, microarray analysis of an *in vivo* model performed by Xu J et al. coincided with our finding that SLC7A11 levels were significantly elevated in mice 6 months after transfection with DNMT3A R882H vectors. As recent discoveries provide similar evidence that the deprived serine/glutamine metabolic pathway is lethal for the growth of leukemia cells [38], we believed that the altered cellular metabolic and increased anti-oxidative stress capabilities might be characteristic for DNMT3A R882 mutant clones.

GSH, an antioxidant in mammalian cells, participates in many important biological processes, especially in cancer cells, such as in carcinogenic mechanisms, cell proliferation, and DNA synthesis, as well as in the regulation of sensitivity against cytotoxic drugs, ionizing radiation and some cytokines [39]. More and more studies have unveiled that in many tumors, chemo-resistance appeared to be associated with higher GSH levels in the cancer cells via GSH conjugation and detoxification [40]. Additionally, our DNR treatment experiment indicated that the apoptotic rate of the mutant clones was significantly lower than that of the wild-type clone and normal K562 cells, which supported our hypothesis that K562 cells with the DNMT3A R882H mutation acquired chemo-resistance via increased levels of cellular GSH. Our results shed new light upon the conclusion drawn by Shlush LI et al. that pre-leukemia HSCs are resistant to induction chemotherapy [36].

In recent studies, researchers reported that SSZ, an inhibitor of SLC7A11, could induce cysteine/cysteine starvation, leading to GSH depletion, which may be useful for therapy against many cancers such as lymphoma [33], prostate cancer [41], breast cancer [42], glioma [43], small cell lung cancer [44] and pancreatic cancer [45]. Therefore, we applied SSZ in our mutant cell model to explore its utility in the treatment of AML patients with the DNMT3A R882H mutation. As a result, we demonstrated that the combined utilization of SSZ with DNR could significantly inhibit the proliferation of mutant clones compared with DNR mono-therapy. Our result confirmed that SSZ could sensitize the mutant clones to DNR via the reduction of intracellular GSH levels, suggesting that in clinical practice, SSZ may be of great value as one ancillary drug for the treatment of AML patients with the DNMT3A R882H mutation. To validate our theory, we further managed block SLC7A11 function by lenti-viral shRNA vector transfection method in order to detect the subsequent GSH level change in all four cell clones. Our results indicated that shRNA induced SLC7A11 blockage significantly decreased the level of GSH in mutated clones compared with wild-type clone and K562, which further emphasized the crucial role of SLC7A11 in intracellular GSH level maintenance, especially in DNMT3A mutated AML clones. Subsequently, colony assay analysis confirmed that SLC7A11 knockdown significantly hampered proliferative abilities of mutated clones comparing with wild-type and K562 clones. However, when DNR treatment is added, all four clones exhibited further decline in colony forming numbers, while clone 43 and clone 160 exhibited insignificant difference comparing with WT clone and K562 cell. This phenomenon was possibly caused by the growth inhibition effect of lenti-viral vector transfection, further experiments of different concentration of DNR combining with shRNA transfection would be needed.

In conclusion, in this study, we successfully established a K562 cell line carrying the DNMT3A R882H mutation by using TALEN technology. Our data revealed that the DNMT3A R882H mutation promoted cell proliferation and enhanced cellular chemo-resistance by elevating intracellular GSH levels. Furthermore, our results indicated that compared with DNR mono-therapy, the combined use of SSZ and DNR promoted greater therapeutic effects on mutant clones, suggesting that in future clinical practice, SSZ could be considered as an ancillary drug for AML patients with the DNMT3A R882H mutation.

## MATERIALS AND METHODS

### TALEN and donor plasmids construction

The DNMT3A gene with the R882H mutation was scanned for potential TALEN-binding sites using free software from the website http://boglabx.plp.iastate.edu/TALENT/ The protocol for the assembly of TALENs targeting DNMT3A was previously described [28]. The module and array plasmids of each TALEN were ultimately constructed into the pcDNA3.1(+) backbone plasmid (V790-20, Invitrogen); the sequences of each TALEN are shown in Figure 1A. The donor plasmid containing the DNMT3A R882H site and homology arms of 1 kb on the 3′ and 5′ ends was constructed using PCR and standard molecular-cloning methods. A 2 kb fragment was amplified by PCR from one patient with the DNMT3A R882H mutation by using the forward primer 5′-CCCCAAGCTTTCCCACCTGACTTGTTTTCC-3′ and the reverse primer 5′-GCCGGAATTCGTCTCCCTGCTGCTAACTGG-3′. Then, the fragment was inserted into the PUC19 plasmid.

### Cell culture, electroporation, sorting, DNA extraction and gene amplification

The K562 cell line was purchased from the ATCC and authenticated at the China Center for Type Culture Collection in November 2014 using short tandem repeat DNA profiling (ABI 3130xl Genetic Analyzer, Life Technologies). K562 cells were cultured in RPMI-1640 (HyClone, Thermo Fisher Scientific) containing 10% fetal bovine serum (Gibco) and 100 U/ml penicillin and streptomycin (Invitrogen) at 37°C in 5% CO2.

To obtain DNMT3A R882H mutant cells, 10 μg of the TALENs pair (5 μg of each plasmid) and donor plasmid (20 μg) were co-transfected into K562 cells by using 4D-NucleofectorTM X Unit (LONZA) with the standard electroporation program FF-120 and SF Cell Line 4D-Nucleofector® X Kit L (LONZA) in a 12-well plate. The cells were collected from the culture plate 72 h post-electroporation to be sorted by FACS for single cell cloning and were then subcultured into 96-well plates.

After the single cell clone expanded, genomic DNA was extracted using a previously described protocol [29]. Genotyping at the TALEN target site was then performed for each sample by PCR amplification (forward primer 5′-TTCTGATTGCTGTGCTTGCT-3′; reverse primer 5′-CCATGTCCCTTACACACACG-3′; 94°C 5 min; 94°C 30 s, 64°C 30 s, and 72°C 1 min for 35 cycles; 72°C 10 min) and Sanger sequencing. Then, the PCR products containing the mutated site were cloned into the pEASY-T1 simple cloning vector (Transgene, China) and sequenced to confirm the mutation site.

### T7 Endonuclease I mismatch detection assay

The T7EI assay was performed as previously described [30]. K562 cells were cultured and electroporated as described above. Genomic DNA was extracted from cells using a QIAamp^®^ DNA Blood Mini Kit (QIAGEN) according to the manufacturer’s handbook.

The fragments (492 bp) of genomic regions that encompassed the TALEN target sites were amplified, purified, melted, and annealed. Then, the hetero-duplex DNA (200 ng) was treated with T7EI (10 units, M0302S, New England BioLabs) for 15 minutes at 37°C and then analyzed on an ABI 3500 Genetic Analyzer (Life Technologies). The primers used to amplify the DNA were the same as primers used above but with 5-FAM modification. To estimate gene modification levels, the area beneath TALEN-specific cleavage peaks (fraction cleaved) was used in the following equation, as previously described: % gene modification = 100 × (1-(1-fraction cleaved)^1/2^) [31].

### Whole-exome sequencing

Exome sequencing was performed as previously described. Generally, genomic DNA from each sample was sheared and ligated to barcoded Illumina sequencing adaptors. DNA was then hybridized using the Roche NimbleGen SeqCap EZ Exome library to capture exomic regions. Exome regions were captured with streptavidin-coated beads and then PCR amplified with Illumina sequencing adaptors. The resulting libraries were sequenced on an Illumina Genome Analyzer IIx or Illumina HiSeq. Reads were mapped to the whole genome using BWA, and a consensus sequence was generated using GATK (Broad Institute best practices). Consensus sequences between the progenitor cell lines and subcloned cell lines were compared to identify candidate novel mutations. Candidate variants that occurred at locations present in the dbSNP database or that showed any presence in the progenitor line were removed. Identified candidate mutations were validated by Sanger sequencing.

### CRISPR vector and single guide RNA (sgRNA) design

Custom-designed synthetic single guide RNAs (sgRNAs) targeting the genomic region covering DNMT3A R882H mutation locus in exon 23 coding sequence (Supplementary Figure 1A) was generated according to the standard procedure described on the website http://www.addgene.org/crispr/zhang sgRNA was cloned into the pKG plasmid which also encodes the Cas9 nuclease, and pcDNA3.1 vector containing the wild-type DNMT3A sequence was used as donor. After successful transfection of the sgRNA vector and donor vector into SKM1 cells, FACS was used to sort single cell expressing GFP into individual wells in 96-well plates and expanded in culture system. Successful mutation by HDR event was assessed by Sanger sequencing (Supplementary Figure 1B).

### Vector transfection and cell sorting

SKM-1 cell line was transfected using 4D-NucleofectorTM X Unit (LONZA) with the standard electroporation program 4D-SF-EH-100 and SF Cell Line 4D-Nucleofector® X Kit L (LONZA) in a 12-well plate. The cells were collected from the culture plate 72 h post-electroporation to be sorted by FACS for single cell cloning and were then subcultured into 96-well plates. After expansion of single cell clone, genotyping of CRISPR target site was performed as previously described.

### Lenti-viral shRNA vector transfection

LV-slc7a11-RNAi vector and hU6-MCS-Ubiquitin-EG-FR-IREs-puromycin negative control vector were purchased from GENECHEM Corporation (Shanghai, China). In detail, LV-slc7a11-RNAi transfected cell express SLC7A11 RNAi sequence, which specifically targeting Slc7a11 mRNA, while hU6-MCS-Ubiquitin-EG-FR-IREs-puromycin vector transfected cell express unrelated control sequence. One day before transfection all four cell lines (K562/ WT clone/ Clone 43/ Clone 160) were incubated overnight in 96-well with density of 4*10^4 cells/well. Next, the cells were cultured with viral supernatant in the presence of 5 μg/ml polybrene for 2 hours. Transfected cells were subject to subsequent experiments after 48 h of culturing.

### Western blot analysis and antibodies

Standard western blot analysis was performed with antibodies for DNMT3A (Cell Signaling Technology, Danvers, MA, USA), SLC7A11 (Abcam, USA) and β-actin (Santa Cruz, CA). The protein bands were detected by enhanced chemiluminescence (Pierce Biotechnology, Rockford, IL).

### Apoptosis assay

After incubation with different chemicals, cells were harvested and stained with fluorescein isothiocyanate (FITC)-conjugated annexin V (annexin V-FITC) and propidium iodide (PI) using the Annexin V-FITC apoptosis detection kit (KeyGen BioTech) according to the manufacturer’s instructions. Then, the cells were analyzed using the FACSCalibur^TM^ system (BD Bioscience). A total of 10,000 events were acquired for each sample, and the data were analyzed using BD CellQuest software.

### Colony forming assay

A total of 1×10^3^ cells were plated in 24-well plate containing 1 ml RPMI-1640 (HyClone, Thermo Fisher Scientific), 20% fetal bovine serum (Gibco), and 0.8% (wt/vol) methylcellulose (Sigma-Aldrich). Colonies comprising 50 cells or more were counted between days 7 and 14, as indicated in individual experiments.

### Chemicals

A sulfasalazine (Sigma-Aldrich) solution was prepared each day in 0.1 M NaOH and was subsequently adjusted with 1 M HCl to a pH of ~8 [40]. Daunorubicin hydrochloride (Sigma-Aldrich) was prepared in DMSO, stored at 20°C, and diluted to suitable concentrations with RPMI-1640 before use. Cytosine β-D-arabinofuranoside (Sigma-Aldrich) was dissolved in RPMI-1640. DL-propargylglycine (Sigma-Aldrich) was dissolved in H2O.

### Gene expression using oligonucleotide microarrays

Total RNA from the cells was extracted, was used in a nonbiased nucleic acid amplification procedure, and was subsequently subjected to oligonucleotide microarray analysis. A GeneChip Human Genome U133 Plus 2.0 Array (Affymetrix) was used for the current study. The analysis software that accompanied the microarray pipeline (Affymetrix® GeneChip® Command Console® Software; Affymetrix) was used to process the data generated from the GeneChips.

### Real-time quantitative reverse transcription PCR

K562 cells and primary cell samples were harvested, and total RNA was extracted using an RNeasy® Mini Kit (QIAGEN). cDNA synthesis was conducted by using a RevertAid RT Kit (Thermo Scientific, USA) according to the manufacturer’s instructions. RT-PCR was performed on a 7900HT Fast Real-Time PCR System (Applied Biosystems) by using the Bestar® SybrGreen qPCR master mix according to the manufacturer’s instructions. The primer sequences used for RT-PCR are depicted in Table 1. Each experiment was performed independently at least three times, and all RT-PCR experiments were performed in triplicate.

**Table 1:** The primer sequences used for RT-PCR

RT-PCR primers			Tm = 59.4°C	
GAPDH	Fw	GACAGTCAGCCGCATCTTCT	Rv	TTAAAAGCAGCCCTGGTGAC
SLC7A11	Fw	TACTGATACTAAATGTTGGCTACCTGTGAT	Rv	GAAGACCCAATAAGTTTGCCGAAGT
CTH	Fw	AGTTGGTGAAGCGTCAGTGTA	Rv	TCTCGGCCAGAGTAAATAGCT
PSPH	Fw	GGAATCATGCGGTGCTGTGAGG	Rv	CCTACGGACGGGCAGGTTCTTAC
PSAT1	Fw	TGACAGGAGCTTGGTCAGCTAAGGC	Rv	TGGGTTGAGGTTCCAGGTGCTTG

### Patients

A total of 41 AML patients admitted to the Department of Hematology at Tongji Hospital of Huazhong University of Science and Technology from July 2008 to November 2016 were recruited in our study. Bone marrow (BM) and/or peripheral blood (PB) cells were collected from these patients using Ficoll density gradient centrifugation (Lymphoprep, Norway) at the time of diagnosis. Informed consent was obtained according to institutional guidelines. Individuals were diagnosed with AML in accordance with the standards of French-American-British classification. The use of human samples was approved by the Ethical Committee of Tongji Hospital of Huazhong University of Science and Technology.

### GSH assay

Cells (2×10^6^ per well) were plated into six-well plates for the assay and were incubated with different chemicals for the appropriate amount of time in an independent experiment. After harvesting the cells, the total intracellular glutathione level was measured using a Glutathione Fluorometric Assay Kit (BioVision, Milpitas, CA) according to the manufacturer’s instructions.

### Statistical analysis

Data, which are presented as the means ± SD of at least three experiments, were analyzed by two-way analysis of variance followed by the Bonferroni test. All p-values were two-sided, and P < 0.05 was considered significant. Statistical analyses were performed with SPSS software (IBM, version 21.0).

## SUPPLEMENTARY MATERIALS FIGURES AND TABLES




