# Supplementary material for: Novel impact of the DNMT3A R882H mutation on GSH metabolism in a K562 cell model established by TALENs
Source: Oncotarget. 2017 Mar 22;8(18):30395–409. doi: 10.18632/oncotarget.16449 (PMC5444751; doi:10.18632/oncotarget.16449)
Supplement: Supplementary file 1 [file oncotarget-08-30395-s001.pdf]

Novel impact of the DNMT3A R882H mutation on GSH metabolism in a K562 cell model established by TALENs

SUPPLEMENTARY FIGURE AND TABLE

A

DNMT-hybrid-f	AAGATTCGGCAGAACTAAGCAG
DNMT-hybrid-r	CCAAACAAACAGAAAACCCC
DNMT-gRNA1up	caccgAGACGTCAGTATAGTGGACT
DNMT-gRNA1down	aaacAGTCCACTATACTGACGTCTC

B

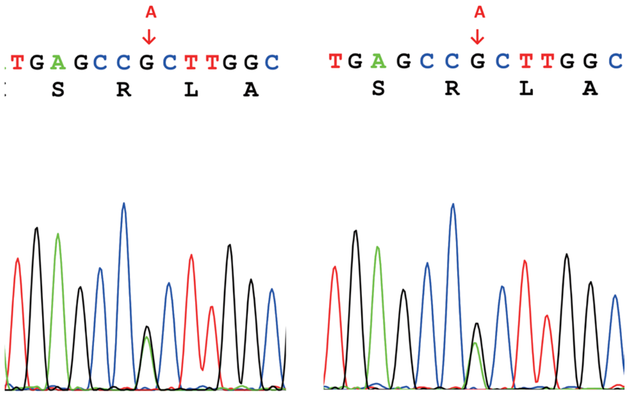

C

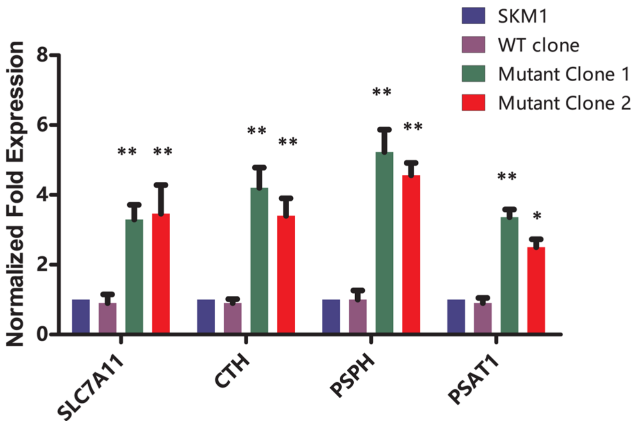

Supplementary Figure 1: (A) sgRNA sequence (DNMT-gRNA1up/DNMT-gRNA1down) and Sanger sequencing primers (DNMT-hybrid-f/DNMT-hybrid-r) for HDR event validation in CRISPR/Cas9 genome editing procedure. **(B)** Sanger sequencing of chromatograms of the generated heterozygous DNMT3A R882H mutated SKM1 cell clones. The mutated site and the corresponding variant were indicated by red arrow. **(C)** RT-PCR validations of target genes SLC7A11, CTH, PSPH and PSAT1 mRNA levels in SKM1 / WT clone/ Mutated Clone 1/ Mutated Clone 2 cell lines. Statistical significance was analysed using two-way ANOVA followed by Bonferroni test (*P<0.05, **P<0.01).

Supplementary Table 1: Potential off-target sites for DNMT3A-TALEN in K562 cell line genome

See Supplementary File 1
